# Pulmonary metastases of a uterine smooth muscle tumor of uncertain malignant potential presenting as growing bullae: a case report

**DOI:** 10.1093/jscr/rjaf106

**Published:** 2025-02-28

**Authors:** Masao Kobayashi, Tomohiro Maniwa, Hisaya Chikaraishi, Hironobu Samejima, Julian Horiguchi, Ryu Kanzaki, Shoji Kamiura, Jiro Okami

**Affiliations:** Department of General Thoracic Surgery, Osaka International Cancer Institute, 3-1-69, Otemae, Chuo-Ku, Osaka 540-0008, Japan; Department of General Thoracic Surgery, Osaka International Cancer Institute, 3-1-69, Otemae, Chuo-Ku, Osaka 540-0008, Japan; Department of General Thoracic Surgery, Osaka International Cancer Institute, 3-1-69, Otemae, Chuo-Ku, Osaka 540-0008, Japan; Department of General Thoracic Surgery, Osaka International Cancer Institute, 3-1-69, Otemae, Chuo-Ku, Osaka 540-0008, Japan; Department of General Thoracic Surgery, Osaka International Cancer Institute, 3-1-69, Otemae, Chuo-Ku, Osaka 540-0008, Japan; Department of General Thoracic Surgery, Osaka International Cancer Institute, 3-1-69, Otemae, Chuo-Ku, Osaka 540-0008, Japan; Department of Gynecology, Osaka International Cancer Institutex, 3-1-69, Otemae, Chuo-Ku, Osaka 540-0008, Japan; Department of General Thoracic Surgery, Osaka International Cancer Institute, 3-1-69, Otemae, Chuo-Ku, Osaka 540-0008, Japan

**Keywords:** bulla, pleural dissemination, pneumothorax, pulmonary metastasis, uterine smooth muscle tumor of uncertain malignant potential

## Abstract

Postoperative recurrences of uncertain malignant potential [(smooth muscle tumors of uncertain malignant potential (STUMPs)] have been reported, with lung as the most common site. Herein, we describe pulmonary metastases of a uterine STUMP presenting as bullae in a female patient. Computed tomography revealed two pulmonary metastases in the left lung with several bullae in both lungs, and the patient was referred to our department for pulmonary metastasectomy. Despite mild right pneumothorax on admission, left pulmonary metastasectomy and bullectomy were safely performed, with an uneventful postoperative course. Right pneumothorax recurred four days after discharge; hence, surgery was indicated. Although the air leak point was not a bulla but rather a pleural dissemination nodule, metastasectomy with bullectomy was performed. Pathological examination suggested that the bullae developed owing to retraction and collapse of the alveoli surrounding the pulmonary metastases. Bullae formed during the clinical course of malignancy have the potential of pulmonary metastases.

## Introduction

Uterine smooth muscle tumors of uncertain malignant potential (STUMPs) are tumors that cannot be clearly diagnosed as either malignant or benign [1]. They do not have high-grade potential; however, recurrence rates have been reported between 8.7% and 11%, and the lung has been identified as the most common site of distant metastasis [2]. Although no standard guidelines for uterine STUMP recurrence have been established, surgical excision of the recurrent nest is the current standard treatment strategy [3].

To the best of our knowledge, this is the first report of pulmonary metastasis of a uterine STUMP forming bullae. We describe a case in which the growth of the bullae was followed up in the clinical course of uterine STUMP, and the resected specimens of bullae were pathologically examined.

## Case report

**Figure 1:**
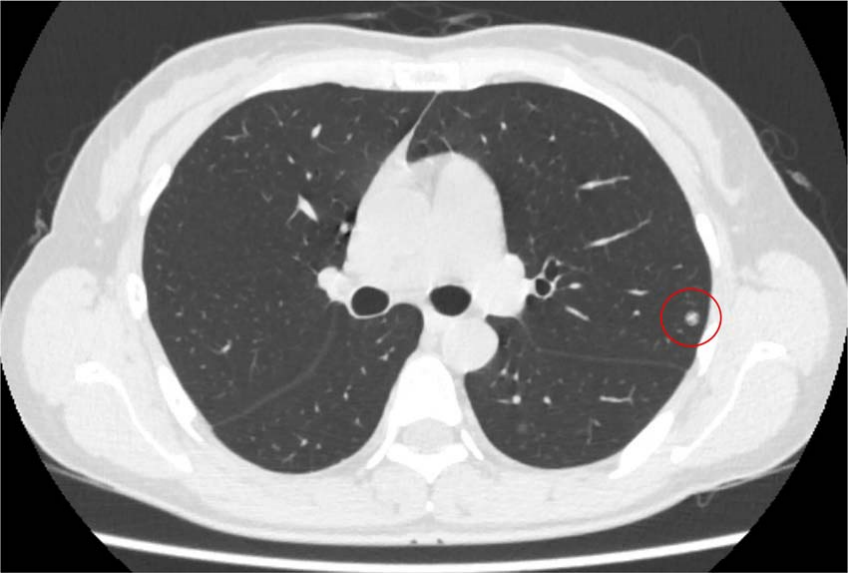
Computed tomography revealed a solitary pulmonary nodule (circle) in the left upper lobe 6 months after uterine surgery.

**Figure 2:**
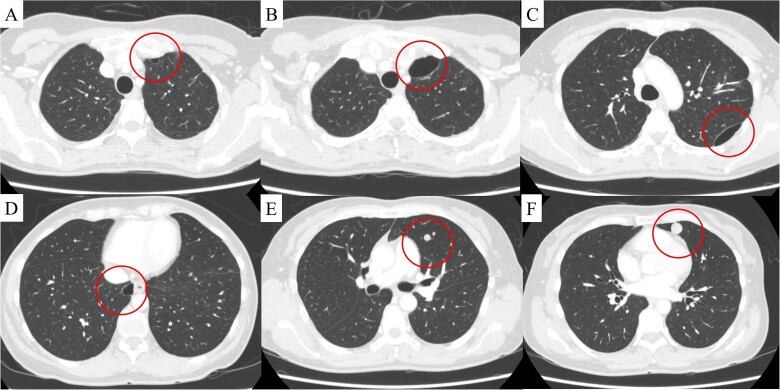
(A) Computed tomography revealed a bulla in the left upper lobe 1.5 years after uterine surgery. At 3 years, in addition to the development of the bulla in the left upper lobe (B), two more bullae appeared and gradually developed in the left (C) and right lower (D) lobes, and two pulmonary metastases appeared in the left lingular segment (E, F). The findings are indicated by circles.

The patient was a 54-year-old woman with no history of smoking. Three years prior to being referred to our department, the patient had undergone total abdominal hysterectomy and bilateral salpingo-oophorectomy for a uterine STUMP. Six months thereafter, a single pulmonary nodule appeared in the left upper lobe (Fig. 1) and pulmonary metastasectomy was performed followed by four courses of gemcitabine-docetaxel therapy. One year thereafter, a single bulla was observed in the left lung (Fig. 2A). Subsequently, several bullae appeared and gradually developed (Fig. 2B–D); at referral, two additional pulmonary metastases were observed in the left lung (Fig. 2E and F). Because no other metastases were present, curative resection of the pulmonary metastases was planned. Additionally, we planned a bullectomy due to the lack of a reasonable explanation for bullae formation.

**Figure 3:**
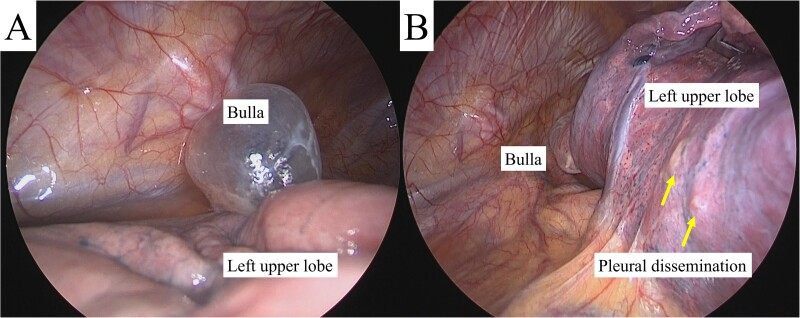
Intra-operative findings revealed a bulla in the apex of the left lung (A) and pleural dissemination (B, arrows).

**Figure 4:**
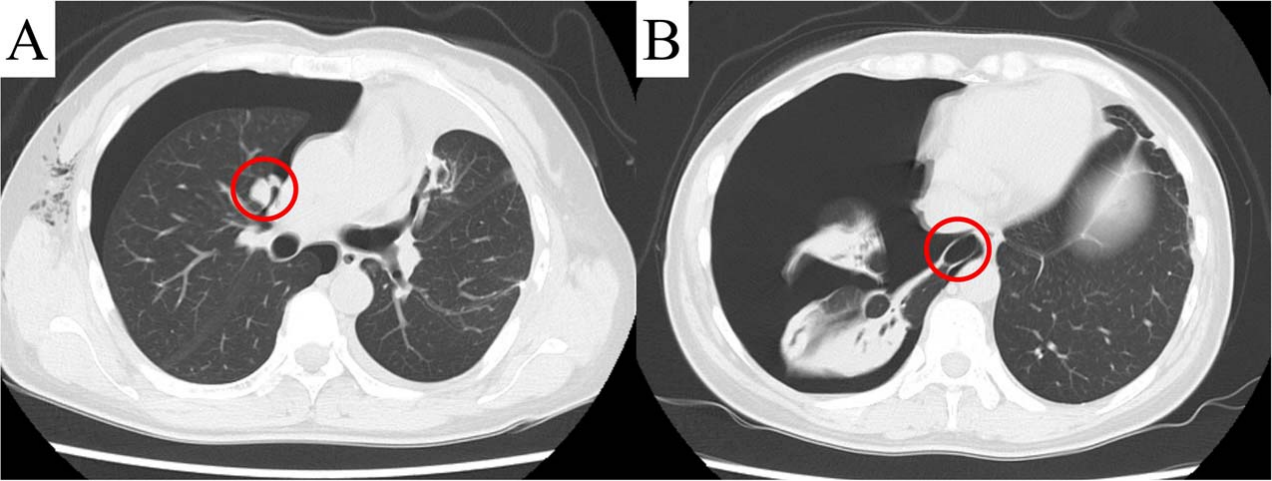
Computed tomography revealed a pleural dissemination nodule in the right hilar area (A) and a bulla in the right lower lobe (B). The findings are indicated by circles.

**Figure 5:**
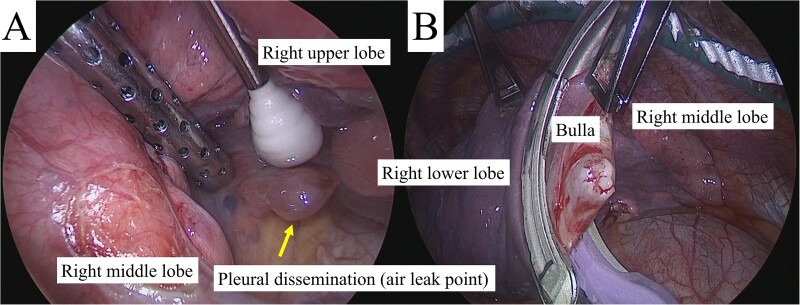
(A) Intra-operative inspection revealed a large pleural dissemination nodule in the right hilar area with an air leak (arrow), which was resected. (B) The bulla in the right lower lobe with no air leak was also resected.

Despite a mild right pneumothorax on admission, the patient underwent a safe operation with chest drainage for the right pneumothorax. Intra-operative findings revealed a bulla at the apex of the left lung and unexpected pleural dissemination (Fig. 3A and B). Therefore, we performed a pulmonary metastasectomy and a pleural biopsy with bullectomy for diagnostic purposes. The patient was discharged without complication or exacerbation of the pneumothorax. However, recurrence of the right pneumothorax was observed four days after discharge, and surgery was scheduled. CT revealed a pleural dissemination nodule in the right hilar area and a bulla in the right lower lobe, in addition to the right pneumothorax (Fig. 4A and B). Intra-operatively, we identified a pleural dissemination nodule in the right hilar area with moderate air leakage, and resection was performed (Fig. 5A). The bulla in the right lower lobe was also resected (Fig. 5B).

**Figure 6:**
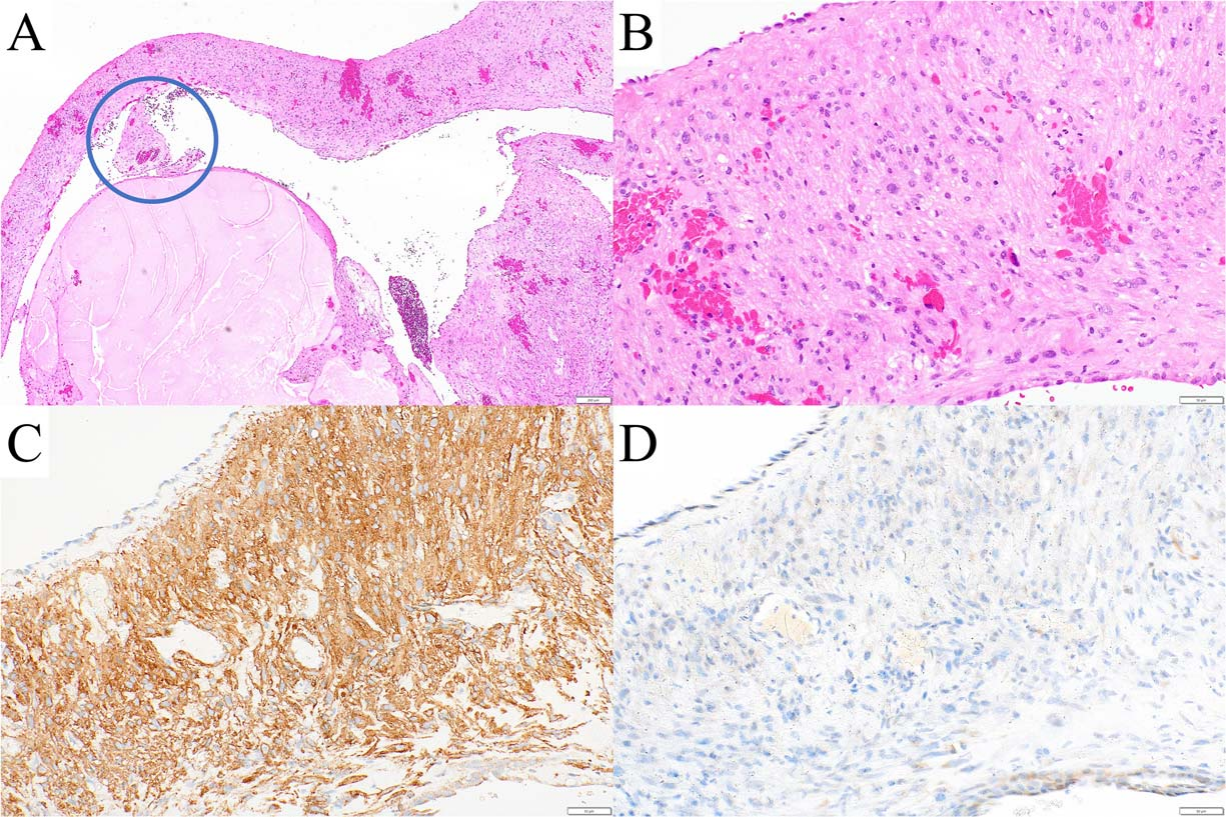
(A, B) Pathological examination revealed tumor cells in the wall of the resected left bulla. A vascular structure was observed in the bulla (A, circle), indicating that the bulla developed by retraction and collapse of the surrounding alveoli. The tumor cells were immunohistochemically (C) positive for smooth muscle actin and (D) negative for human melanoma black 45, ruling out LAM and Birt–Hogg–Dubé syndrome.

Pathological examination revealed that the pulmonary and pleural nodules were metastases of the uterine STUMP. Furthermore, the wall of the resected left bulla contained tumor cells (Fig. 6A and B). Vascular structures were identified in the resected left bulla (Fig. 6A), indicating that the bulla developed due to retraction and collapse of the surrounding lung. Pathological findings of the right bullae exhibited similar features. Diagnoses of lymphangioleiomyomatosis (LAM) or Birt–Hogg–Dubé syndrome were ruled out owing to immunohistochemical positivity for smooth muscle actin and negativity for human melanoma black 45 (Fig. 6C and D). No recurrent pneumothorax was observed after surgery, and doxorubicin was administered 3 weeks later.

## Discussion

**Figure 7:**
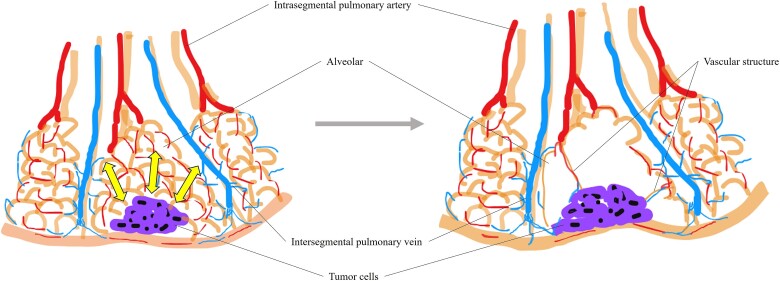
Tumor cells reduced the flexibility of the surrounding alveoli, causing local hypertension and traction (arrow). The mechanical force destroyed some alveolar walls and led to bulla formation, while more robust vascular structures survived within the bulla.

Although no large-scale studies of pulmonary metastasis of uterine STUMPs have been reported, the multiple nodular patterns in both lungs are common [4]. We identified limited detailed reports of pulmonary metastases of uterine STUMP [5–8], and none describe bulla formation caused by metastases. Several mechanisms of bulla formation associated with pulmonary malignancy have been proposed [9]. Further, bullae have been reported to develop by retraction of the pulmonary alveoli [10]. In the present case, vascular structures were observed within the bullae, suggesting that bullae formed through a distinct mechanism. Compared with vascular structure, the alveolar wall is more fragile and easily ruptured by mechanical traction. In addition, pulmonary metastasis may decrease the flexibility of the surrounding alveoli. The pathological findings in the present case suggest that the fragile alveolar wall was ruptured by local hypertension due to pulmonary metastasis (Fig. 7). As differential diagnoses, LAM and Birt–Hogg–Dubé syndrome are lung cyst-forming diseases that occur in middle-aged women [11]. However, the pathological assessment of the bullae excluded these conditions.

Uterine STUMP typically has a good prognosis, and long-term survival has been reported even after recurrence [5]. Nevertheless, Kotsopoulos *et al*. described a case of multiple pulmonary metastases and pleural dissemination with a poor prognosis, in which the patient died 11 months after diagnosis, despite chemotherapy [8]. In the present case, we aimed to improve the patient’s prognosis by resecting the pulmonary metastasis. However, complete resection of the metastases was impossible due to pleural dissemination, and systemic chemotherapy was initiated. Although no established chemotherapy protocol exists, a combination of doxorubicin and cisplatin is the most commonly used regimen [5, 12]. Recently, the efficacy of letrozole and immunotherapy have been reported [13, 14], and further research is required to establish an optimal therapeutic strategy.

As a study limitation, positron emission tomography/computed tomography (PET/CT) was not performed. PET/CT is invaluable for detecting uterine sarcomas [15] and may have detected the bullae caused by pulmonary metastases earlier, allowing for more timely surgical intervention. Given uncommon patterns of pulmonary metastases, as observed in our case, and the invasiveness of surgery, the treatment should be deliberated.

In summary, we report a novel case of growing bullae caused by pulmonary metastasis of a uterine STUMP. A pathological examination clarified that the bullae were formed by retraction and collapse of the surrounding alveoli. We consider this case to be rare and valuable for a better understanding of the formation of bullae during the clinical course of malignancy.
